# Effects of Housing and Management Factors on Selected Indicators of the Welfare Quality^®^ Protocol in Loose-Housed Dairy Cows

**DOI:** 10.3390/vetsci9070353

**Published:** 2022-07-13

**Authors:** Daniel Gieseke, Christian Lambertz, Matthias Gauly

**Affiliations:** 1 Department of Animal Sciences, Georg-August-University, 37075 Göttingen, Germany; 2 Faculty of Science and Technology, Free University of Bolzano, 39100 Bolzano, Italy; christian.lambertz@fibl.org (C.L.); matthias.gauly@unibz.it (M.G.)

**Keywords:** animal welfare, dairy cow, housing, management, Welfare Quality^®^ protocol

## Abstract

**Simple Summary:**

Housing conditions and management practices affect animal welfare levels in livestock production. The objective of this study was to investigate potential effects of housing and management factors on animal welfare in dairy cattle by comparing different farms with each other in a benchmarking approach. For this purpose, 63 dairy cattle farms in Northern Germany were assessed using an animal welfare indicator system (Welfare Quality^®^ protocol). Farms were categorized into a high and a low welfare group for each of the selected animal welfare indicators (body condition score, integument alterations, lameness, milk somatic cell count, and social behaviour). Both groups were compared regarding housing conditions and management practices in a statistical analysis. Clear differences between the groups were found for lameness concerning the routine use of foot-baths, milk somatic cell count concerning the milking frequency, and social behaviour concerning the cow-to-stall ratio. Comparing farms with high and low animal welfare status regarding housing and management factors provide useful information for the practice. Dairy cattle farmers could use these findings to improve animal welfare on their farms.

**Abstract:**

The objective of this study was to examine the effects of housing and management factors on animal welfare indicators in dairy cows using a benchmarking approach. In total, 63 conventional dairy cattle farms with zero-grazing in Northern Germany were assessed using selected animal welfare indicators (body condition score, integument alterations, lameness, milk somatic cell count, and social behaviour) of the Welfare Quality^®^ protocol. Additionally, housing characteristics such as designs of barns, cubicles, and floors were documented during farm visits and farmers were interviewed concerning their common management routines. Farms were categorized into a high welfare or low welfare group by calculating upper and lower tertiles for each of the animal welfare indicators separately. Both groups were compared regarding housing conditions and management practices using univariable and multivariable logistic regressions. Several associations between housing and management factors and animal welfare indicators were demonstrated in univariable analysis (*p* < 0.20). Significant effects within multivariable logistic regression analysis were determined for lameness (routine use of foot-baths), milk somatic cell count (milking frequency) and social behaviour (cow-to-stall ratio) (*p* < 0.05). Comparing farms with higher and lower animal welfare status can provide useful information about effective options to improve animal welfare.

## 1. Introduction

Housing conditions (e.g., design of feeding, resting, and walking area) are highly relevant for the animal welfare of intensively housed dairy cattle, because they spend most of their lifetime indoors [1,2,3]. In several studies different effects of housing conditions on single animal welfare indicators were examined. For example, Dippel et al. [4] described a relationship between flooring design and the prevalence of lameness (*p* < 0.05). The risk for lame cows was higher in farms with slatted floors (Odds Ratio (OR) = 1.3), compared to farms with solid floors (OR = 1.0) [4]. In contrast, Solano et al. [5] estimated no effect of the flooring type on lameness prevalence. Cubicle design can also affect the behaviour and health of dairy cows. Higher standing times in cubicles and lower numbers of stall use sessions for cows were found in cubicles with mattresses, compared to those with deep bedding cubicles [6]. The latter are also associated with a lower risk of prevalence and severity of hock lesions, due to the softer lying surface [7]. For example, lower risks of hock lesions were found in cubicles with deep bedding, compared to cubicles without deep bedding [8]. In addition to the housing conditions in the barns, management decisions of the farmers can also affect the animal welfare level of dairy cows, directly or indirectly. Wearing gloves during milking occurs more likely in herds having lower bulk milk somatic cell counts < 400,000 cells/mL [9], and using coliform mastitis vaccine reduces high bulk milk somatic cell counts [10]. Furthermore, routinely administered antibiotics during the dry period decreased the risk of subclinical mastitis in Swiss dairy farms [11]. Management decisions of the farmers can also have an impact on the behaviour of dairy cows. For example, the average frequency of displacements was negatively associated with continuous availability of roughage and introducing heifers before calving in the lactating group [12]. Similarly, overstocking (i.e., raising more cows than stalls or feed bunk spaces) leads to higher displacement rates at the feed rack [13] and prolonged standing times in the walking alleys [14]. Most studies examined the influence of housing conditions and management on one or more animal welfare indicators by conducting a risk factor analysis. Dairy farms with lower animal welfare levels regarding lameness e.g., [4,15], hock lesions e.g., [16,17], mastitis e.g., [10,11] or displacements [18] were compared with larger control groups. The discriminatory power between both groups might have been relatively small due to this study design and consequently potential influencing factors may have been undetected. The objective of the present study was to compare dairy farms showing larger variations within selected animal welfare indicators (body condition score, integument alterations, lameness, milk somatic cell count, and agonistic interactions) using a benchmarking approach. The results of the study could contribute to increase the scientific knowledge of associations between housing and management factors and selected animal welfare indicators (cognitive goal). Furthermore, the newly developed benchmarking approach can be applied in the future in agricultural practice to optimize animal welfare in dairy farming (utilitarian goal). This method might help farmers to identify main animal welfare problems and motivate them to improve housing and management in their herds due to information on best practices and peer comparison [19,20]. For this purpose, dairy farms were categorized as high welfare groups (HW) and low welfare groups (LW) by calculating upper and lower tertiles for each animal welfare indicator of the Welfare Quality^®^ protocol (WQP) [21] separately and compared with regard to their housing conditions (barn, cubicle, and floor design) or management practices.

## 2. Materials and Methods

### 2.1. Study Design

Data collection was conducted from October 2014 to September 2016 by one intensively trained assessor on 63 conventional loose housing dairy cattle farms with zero-pasturing located in Northern Germany. The animal welfare level was assessed applying the complete WQP. This is a standardized indicator system for on-farm animal welfare assessment. It focuses mainly on animal-based measures, which directly reflect the actual welfare state of the animals. More than 30 animal welfare indicators from the fields of feeding, housing, health and behaviour were measured and aggregated to 12 welfare criteria and 4 welfare principles [21]. Five indicators of the WQP (body condition score, integument alterations, lameness, milk somatic cell count, and agonistic interactions) were selected in the present study for testing the new benchmarking approach as they reflect diverse aspects of animal welfare (feeding, health, and behaviour) and because variations between farms were greatest for these indicators. High variability between dairy farms indicate potential influencing effects of housing and management factors.

### 2.2. Farm Selection

Farm acquisition was organized with the support of different agricultural stakeholders (e.g., chamber of agriculture, milk recording association, and research facilities). Farms were selected as part of a research project on the influence of herd size on animal welfare, so that the number of cows per farm was higher than the national average [22]. Comparability of the housing environment between farms should be given, therefore they had to fulfil specific requirements for participating in the study. The lactating cows should be all kept in loose housing barns with deep bedded or rubber mat-equipped cubicles. Only farms with Holstein Friesian as the dominant breed were permitted in order to exclude genetic effects. Due to the high weighting of the resource-based indicator access to pasture within the aggregation system of the WQP, only farms with less than six hours pasture access were accepted for the study. There were no other limitations regarding housing conditions, milking techniques or feeding systems (for further characterizations of the farms see Table 1).

### 2.3. Data Collection

The assessment of animal welfare indicators was carried out in accordance with the requirements of the WQP for dairy cattle [21]. For practical reasons, non-lactating cows and cows in hospital pens were excluded from the sample. Descriptions of the animal welfare indicators and assessment methods executed on the 63 dairy farms can be found in Table 2. Data collection was performed at each farm in a fixed order. At the beginning of the farm visit, agonistic interactions between cows were recorded in up to 12 different segments of the barn using continuous behaviour sampling. Subsequently, clinical examination of individual cows was conducted on a sample depending on herd size (range: 32 to 102 cows). The indicators were each assessed on the same sample of animals (body condition, integument alterations, and lameness). Finally, milk-recording data (milk somatic cell count (SCC)) of the last three months prior to the farm visit were requested in a farmer interview. In addition to the indicators of the WQP, farm characteristics such as barn, cubicle, and floor design or feeding system were recorded according to von Keyserlingk et al. [23]. Cubicle dimensions including bed length from curb to brisket locator, neck rail distance from the rear curb, neck rail height from the bedding, and average distance from stall partition to bedding were recorded as examples on 2 to 5 stalls in each dairy farm (depending on the uniformity of the stalls). Stall width was measured as the distance between two adjacent stall partitions on at least 10 stalls, because stall width differed more frequently compared to other stall dimensions. Double-row cubicles and cubicles against walls were surveyed separately and average stall dimensions were weighted according to their presence in the different pens. Type of stall type (deep bedded, rubber mat), presence of brisket locator, flooring type (slatted floor, solid floor), presence of rubber mats on the floors, type of feeding barrier (neck rail, feed racks), stall climate equipment (curtains, ventilators), barn equipment (concentrate feeder, cow brushes) or barn construction (insulated, non-insulated) were recorded through direct observation. Cow-to-stall ratio was calculated by counting animals and stalls in each group of lactating dairy cows (>100% = overstocking; <100% = understocking). Cow-to-feeding place ratio was calculated by assessing the number of feed racks and the number of dairy cows per group. In farms with neck tubes, length of the feed alley was measured and divided by 0.6 m to estimate the cow-to-feeding place ratio as proposed by von Keyserlingk et al. [23]. Surfaces of walking alleys, feed alleys, crossovers and, if appropriate, loafing yards were summed up and divided by the number of dairy cows per group to calculate the provided walking space abilities (m^2^). Trough lengths as well as average widths of alleys and crossovers were measured separately for every pen. Recorded values were weighted by the number of dairy cows in each lactation group, because housing conditions and group sizes partly differed within the farms. Management practices from the fields of feeding management (e.g., amount of concentrates per cow, feeding frequency, body condition scoring), cleaning management (e.g., cleaning frequency of cubicles, walking alleys), milking procedures (e.g., milking frequency, milking system, post-dip) or health management (claw trimming, claw bath, dry cow therapy with antibiotics) were obtained through farmer interviews.

### 2.4. Statistical Analysis

Prevalence of the selected welfare indicators (body condition score, integument alterations, lameness, milk somatic cell count, and agonistic interactions) were dichotomized by calculating tertiles. The upper tertile of the 63 dairy farms represents higher welfare, and the lower tertile of the dairy farms represents lower welfare within each indicator. The remaining farms of the medium tertile were not considered in statistical analysis in order to improve the discriminatory power. The indicators of body condition score, integument alterations, lameness, and milk somatic cell count were expressed as percentage of affected cows (%). The welfare indicator agonistic interactions (head butts/displacements per cow/hour) were aggregated to the criterion “social behaviour” using I-spline-functions as described in the WQP. Criterion scores are expressed on a 0 to 100 scale (0 = poor, 100 = good). Accordingly, upper and lower tertiles were calculated with the respective scores. All statistical analyses were computed with SAS, version 9.4 (Statistical Analysis Systems, Cary, NC, USA). Dairy farms with higher and lower welfare levels within each indicator (response variable) were compared regarding several housing and management factors (predictor variables) using a logistic regression analysis (Proc logistic). Continuous housing (e.g., stall dimensions, trough length) and management (e.g., amount of concentrates, cow-to-stall ratio) variables were categorized using tertiles. For each animal welfare indicator several housing and management factors were selected as potential predictors. The preselection process was based on assumed causal associations between response and predictor variables found in the literature or own observations during farm visits. First, collinearity between all housing and management factors was checked using Pearson’s Chi-square test of independence. Phi Coefficient or Cramer’s V (depending on the amount of categories) were calculated to assess the effect size. The threshold for collinearity was set at 0.80, but no variable combination within the data set exceeded this value. Each preselected housing and management factor was analysed individually with a logistic regression analysis, in order to identify associations between predictor and response variables. OR and 95% confidence intervals were calculated to describe the probability of each herd being in the higher welfare group. All predictor variables with *p* < 0.20 were included in subsequent multivariable statistical analyses. The final logistic regression models for each welfare indicator were fitted using automated stepwise selection procedure. The statistical procedure adds or removes variables to the model considering a significance level of *p* < 0.05. Standardized deviance residuals were examined visually.

## 3. Results

Characterization of dairy farms with higher or lower animal welfare levels of specific indicators of the Welfare Quality^®^ assessment are shown in Table 3. Based on the calculation of tertiles from the results of the welfare assessments, farms were classified as having lower welfare levels when they exceeded the threshold of 15.6% lean cows. In contrast, farms with less than 9.2% lean cows were classified as having higher welfare levels. On average of all studied farms, 13.0% lean cows were determined. In group HW 6.3% (range 0.0 to 9.1) lean cows were found, and 19.9% (15.9 to 29.2) were determined in group LW. The mean percentages of lesions and swellings were 16.5% (6.3 to 27.1) in group HW and 47.4% (38.6 to 62.7) in group LW, respectively (overall mean: 32.1%). Farms of group HW had on average 11.9% (0.0 to 21.9) severely lame cows, whereas 52.9% (37.5 to 74.3) were detected in group LW (overall mean: 31.8%). In HW on average 13.0% (2.3 to 17.6) of the cows had a somatic cell count of >400,000 cells. In the LW group the value was 27.6% (22.8 to 37.4). On average of all farms, 20.1% affected cows were found. Farms with higher welfare levels within the criterion “social behaviour” had average scores of 92.6 points (90.1 to 96.6). In contrast, farms of group LW had on average 71.6 points (40.4 to 83.0). The average score in the total sample was 83.8 points. 

### 3.1. Body Condition Score

In total, 10 preselected housing and management variables were considered as potential influencing factors in univariable statistical analysis. Four variables were associated with being a high welfare level herd, indicating lower percentages of lean cows (Table 4). Higher feeding frequencies (>1 v. 1 time/day), feeding total mixed ration (v. partly mixed ration), several feeding groups (v. 1 feeding group) and the amount of concentrate feeding (>8.5 v. <8.5 kg/day) were positively associated with lower percentages of very lean cows (*p* < 0.20) and therefore included in subsequent statistical analyses. None of the tested variables was significant in the multivariable logistic regression analysis (*p* > 0.05).

### 3.2. Integument Alterations

Of the 10 preselected housing and management variables, none were associated with a high welfare level herd, indicating lower percentages of cows with severe integument alterations such as lesions or swellings (*p* > 0.20) (Table 5). Therefore, no subsequent multivariable statistical analyses could be conducted and no significant effects could be determined in a final model.

### 3.3. Lameness

Of the 11 preselected housing and management variables five were associated with HW, indicating lower percentages of severely lame cows (Table 6). Medium cow-to-stall ratio (95–105% v. <95% and >105%), high frequency of claw trimming (>2.5 v. <2.5 times/year), routine use of footbaths (v. no routine use), solid floors (v. slatted floors) and presence of rubber mats on the floor (v. no presence of rubber mats) were positively associated with lower percentages of severely lame cows (*p* < 0.20) and therefore included in subsequent statistical analyses. Routine use of footbaths was significant in the multivariable logistic regression analysis (*p* < 0.05). The final model reached a coefficient of determination of r^2^ = 0.10. 

### 3.4. Milk Somatic Cell Count

Three of the 12 preselected housing and management variables were associated with being a high welfare level herd, indicating lower percentages of cows with high milk somatic cell counts (>400,000 cells/mL) (Table 7). Milking in a milking parlour (v. automatic milking systems), lower milking frequency (2 v. >2 times/day) and routine dry cow antibiotic therapy (v. on demand) were positively associated with lower percentages of cows with increased cell counts (*p* < 0.20) and therefore included in subsequent statistical analyses. Frequency of milking was significant in the multivariable logistic regression analysis (*p* < 0.05). The final model reached a coefficient of determination of r^2^ = 0.13.

### 3.5. Social Behaviour

Seven of the 12 preselected housing and management variables were associated with HW, indicating lower agonistic interactions between dairy cows (Table 8). Integration of heifers in the herd before calving (v. after calving), lower cow-to-stall ratios (<95% v. >95%), higher feeding alley widths (>3.6 m v. <3.6 m), higher walking spaces per cow (>4.4 m^2^ v. <4.4 m^2^), presence of concentrate feeder stations (v. absence), medium cow-to-feeding place ratios (105–118% v. <105% and >118%) and medium trough length per cow (4.7–6.8 cm v. <4.7 and >6.8 cm) were positively associated with lower agonistic interactions between dairy cows (*p* < 0.20) and therefore included in subsequent statistical analyses. Cow-to-stall ratio was significant in the multivariable logistic regression analysis (*p* < 0.05). The final model reached a coefficient of determination of r^2^ = 0.18.

## 4. Discussion

### 4.1. Limitations of the Study

The objective of the present study was to compare housing and management factors of dairy cattle farms showing larger variations within selected animal welfare indicators (body condition score, integument alterations, lameness, milk somatic cell count, and agonistic interactions) of the WQP [21]. This animal welfare indicator system is only suitable to a limited extent for use in agricultural practice, as it is very time-consuming and cost-intensive [25,26]. However, the newly developed benchmarking approach might also be applied with other animal welfare assessment methods, which are more common in practice e.g., [27]. Furthermore, a combination with precision livestock farming technologies is also conceivable to reduce the duration of the welfare assessments [28]. Practical guidelines and official recommendations can also be used as a reference point to improve animal welfare in dairy cattle farms. For example, de Boyer des Roches [29] investigated if compliance with design recommendations for cubicles and self-locking barriers was associated with skin alterations, dirtiness and lameness. Similarly, Gaworski and Bockowski [30] proposed a method by which farms can compare themselves in terms of their fulfilment with national recommendations in different zones of the barn (lying, social, feeding, and milking areas). Perhaps a combination of the benchmarking approach and other tools considering recommendations could increase animal welfare levels on dairy cattle farms. This should be investigated in further studies. The benchmarking approach was chosen because in a classical risk factor analysis with small experimental and larger control groups, the discriminatory power between both groups compared might be relatively small. Consequently, potential influencing factors of housing and management on animal welfare may remain undetected. Therefore, 63 dairy cattle farms with low and high animal welfare levels regarding several animal welfare indicators were examined in the present study. The farms were selected for a larger study on the influence of herd size on animal welfare, so that the mean number of animals per farm (368 cows) was significantly higher than the national average (61 cows) in Germany [22]. However, it can be considered representative given that (1) several farm types (e.g., tie stalls, straw-bedded barns, and farms with pasture access), which generally are known for lower herd sizes, were excluded in the present study and (2) the intensification of milk production continues with further rising herd sizes. Distribution of specific housing conditions (e.g., cubicle types, flooring types and stall climate) was not considered during farm acquisition as no statistical data concerning their proportions were available to compile a representative sample. Nevertheless, housing conditions and management practices can be considered as typical for zero-grazing free stall dairy farms. 

### 4.2. Comparison of Prevalences

The percentages of the most examined animal welfare indicators (except lameness) and scores of the welfare criterion “social behaviour” were in accordance with the findings of other studies using the WQP. For example, Popescu et al. [31] observed on average 13.1% lean cows in loose houses, and Zuliani et al. [32] found 18.3% cows with a lower body condition score in Italian mountain farms. Lower median percentages of lean cows (9.1%) were found in a French study by de Boyer des Roches [33], whereby visited farms showed a wide range (0.0 to 87.5). Higher prevalences (33.0%) were determined by Benatellah et al. [34] in Algerian dairy farms. The observed percentages of cows with severe integument alterations (lesions/swellings) in the present study comply with the mean prevalence of 39.2% in French [24], 37.6% in Dutch [35] and 29.8% in British [26] dairy cattle farms. Lower percentages of lesions and swellings were found in Italy (12.0%) with a wide range (0.5 to 26.2) between the farms [32]. The prevalences of severely lame cows in the present study are on a higher level compared to other examinations using the WQP. Coignard et al. [24] found in France only 2.9% (0.0 to 34.6) severely lame cows, and de Vries et al. [35] detected 5.0 % (0.0 to 65.9) in the Netherlands. A British study revealed a similar result of 4.9% (0 to 47.6%) severely lame cows [26]. Slightly higher prevalences were observed with 7.5 % (0.0 to 22.0%) in a recent study [36]. Conceivably, lameness prevalence in the present study was influenced by a combination of several risk factors [4]. Straw barns, tie stalls and pasture systems were excluded from the study, which are known as beneficial for preventing claw disorders or lameness [15,37,38]. Percentages of cows with high milk somatic cell counts were in accordance with other studies. Coignard et al. [24] observed on average 20.6% dairy cows with a SCC > 400,000 cells (2.0 to 46.6%). The prevalences were somewhat lower with 11.1% in the Netherlands [35], 15.1 % in Germany [39] and 15.5% in the United Kingdom [26]. The number of head butts and displacements per cow and hour were at a comparable level to previous findings. For example, Andreasen et al. [40] reported that all farms achieved high scores in this criterion due to the low occurrence of agonistic interactions between the cows. In a Belgian study de Graaf et al. [41] calculated on average a score of 77.0 (55.2 to 91.2) at the beginning and 76.0 (56.8 to 98.4) at the end of the indoor season. Slightly higher scores were found with 83.4 points [39] and 94.0 points [36] in German dairy cattle farms.

### 4.3. Body Condition Score

Farms with higher feeding frequencies, i.e., providing fresh feed more than once per day were potentially at a lower risk of having poor body condition scores (*p* < 0.20). These findings are in accordance with DeVries et al. [42], who observed a positive effect of higher (2 times/day) compared to lower (1 time/day) feeding frequencies on the feeding behaviour of dairy cattle. The authors reported that dairy cows increased their daily feeding time and subordinate cows were less frequently displaced at the feed rack [42]. Additionally, higher feeding frequencies could reduce the amount of sorting for grain concentrate components [43]. This behaviour can lead to a dilution of the ration and consequently results in an insufficient uptake of nutrients, particularly for subordinate cows which are often forced to consume feed remains [44,45,46]. HW farms frequently had more than one feeding group (i.e., providing at least two different rations), compared to LW farms. Establishment of different feeding groups allows the farmers to adjust the rations for specific feeding requirements in particular lactation stages. Dairy cows of different feeding groups receive concentrates according to their milk yield and the risk of under- or overconditioning is therefore reduced [47,48,49]. Provision of high amounts of concentrate feeds (>8.5 kg/day) predictably reduced the risk for low body condition score. Similarly, Berry et al. [50] found heavier cows with smaller weight losses during lactation in groups with high energy feed levels. Nevertheless, higher amounts of concentrate feeds might contribute to overconditioning of the dairy cows in the late lactation stages. Fat cows more often develop metabolic disorders such as ketosis, which is caused by an increased mobilization of fat in early lactation [51]. However, none of the associated housing and management variables of the univariable analysis were significant in the multivariable logistic regression.

### 4.4. Integument Alterations

None of the preselected housing and management variables affected the probability for cows with fewer lesions or swellings (*p* > 0.20). These findings were unexpected, because the prevalence of severe integument alterations is mainly influenced by housing and management conditions [48,52,53]. Cubicles with rubber mats seemed to be a risk factor for hock lesions, because the cows lie on a hard and abrasive surface [8,37]. Wechsler et al. [54] determined higher incidences of hairless patches, scabs or wounds in cubicles with soft lying mats, compared to cubicles with straw bedding (*p* < 0.05). Positive effects of deep-bedded cubicles concerning severe integument alterations were also found in Germany and Austria [36,55]. Furthermore, impacts of cubicle size on the prevalence of integument alterations were reported in several studies [7]. For example, Potterton et al. [56] detected fewer hock swellings, if the distance between curb and brisket board exceeded 178 cm. Kielland et al. [57] determined a lower risk for hock injuries, if the diagonal free stall length (i.e., distance from neck rail to rear curb) was larger than 196 cm, compared to shorter distances (≤196 cm). Cows in herds with recommended cubicle widths had lower risks (OR = 0.36) for severe hock lesions than cows in smaller cubicles in Swedish dairy cattle farms [58]. Fewer swellings (OR = 0.6) were observed if cows were not in contact with rails while rising in a British study [56]. Besides the impact of the lying area on integument alterations, other barn equipment might also influence the number of cows with lesions or swellings (e.g., feeding rack type, feeding rack height). Zaffino Heyerhoff et al. [59] observed higher risks of developing neck injuries in farms with lower (≤149 cm), compared to farms with higher feed rail heights (>149 cm). However, contrary to expectations none of the preselected housing and management variables were associated in the univariable logistic regression. Conceivably, management factors (e.g., frequency of litter or type of bedding material) were more important for the frequency of integument alterations than housing environment alone [7,20,58]. Additionally, the distribution of some potential influencing factors (e.g., rubber-mat v. deep-bedded cubicle) in the sample was not balanced. This may have contributed to the lack of significant effects in the present study. Both aspects should be considered in future research.

### 4.5. Lameness

Dairy farms with higher cow-to-stall ratios (>105%) had a higher risk of severely lame cows, compared to dairy farms with lower cow-to-stall ratios. These findings might be explained by the changing lying and standing behaviour under overstocked conditions [14,15,60]. For example, Falk et al. [61] detected higher standing times in the alleys with increasing cow-to-stall ratios due to waiting times for vacant cubicles. Prolonged standing in the soiled walking alleys and crossovers can negatively affect claw health. Contact with manure might lead to chemical exposure on sole and heel, resulting in claw disease and subsequent lameness [14,62]. Higher claw-trimming frequencies (>2.5 times/year) were associated with lower risks of severely lame cows, compared to infrequent claw trimming. These findings were similar to Espejo and Endres [15] and Oehm et al. [63], who stated a positive effect of higher hoof-trimming frequencies on the lameness prevalence in high-producing dairy cattle (*p* < 0.05). In contrast, Chapinal et al. [64] and Blanco-Penedo et al. [53] did not determine an association of hoof-trimming frequency on lameness in dairy cattle. Farms with slatted floors had a higher risk of severely lame cows, compared to farms with solid floors. Similarly, Dippel et al. [4] observed higher lameness prevalences in Austrian dairy cattle farms in slatted (OR = 1.3), compared to solid (OR = 1.0) floor. Rouha-Mülleder et al. [65] also proved higher percentages of lame cows in farms with slatted floors. The authors stated that lameness prevalence increases due to protruding ridges or differences in the contour lines [4,65]. No association of flooring type on the number of lame cows was determined by Solano et al. [5]. Results of the present study showed an effect of routine usage of footbaths on the number of severely lame cows in the multivariable analysis (*p* < 0.05). Footbaths were routinely used by 95% of the farms within group HW, compared to only 43% by those in the group LW. Similarly, an associated decrease in lameness prevalence with higher footbath frequencies was found by Chapinal et al. [64] and Griffiths et al. [66]. No association between footbath usage and lameness prevalence was examined by Espejo and Endres [15] and Adams et al. [48]. This might be explained by different causes of lameness [66]. Footbaths are beneficial for preventing infectious diseases through cleaning of the claws [67], but may not help to improve other claw disorders [15,64]. Major differences in footbath design and application protocols may have contributed to the conflicting results [68].

### 4.6. Milk Somatic Cell Count

Herd managers of the LW group (80%) used routine antibiotic dry cow therapy more frequently, compared to those of the HW group (52%). The observed association was in accordance with the results of a case-control study in Sweden [69]. The authors stated that dairy cows of herds with high proportions of subclinical mastitis were treated more frequently compared to those with a low prevalence of subclinical mastitis [69]. However, these results indicate probably no causal relationship between routine antibiotic dry cow therapy and higher mastitis incidence [70]. Farms with higher percentages of cows with increased milk somatic cell count have a higher risk of udder infections during the dry period due to increased udder infections at dry off and a likely higher number of contagious pathogens [71]. Conceivably, the farmers of the LW group might have tried to eliminate existing intramammary infections and prevent further udder infections applying routine antibiotic dry cow therapy, while farmers of the HW group were more often able to reduce antibiotic administration and use internal teat sealants as alternative protective measure [72,73]. In contrast, Doherr et al. [11] reported lower risk of subclinical mastitis with routine use of antibiotic dry cow therapy (OR = 0.5), compared to infrequent antibiotic treatments (OR = 1.0). More frequent milking (>2 times/day) was positively associated with higher milk somatic cell counts (*p* < 0.05). These findings were unexpected, because increased milking frequency is usually associated with lower milk somatic cell count levels due to more frequent discharging of the udder [74]. Lower percentages of cows with high milk somatic cell counts were detected in herds with three, compared to herds with two milking times by Smith et al. [75]. This contradictory result might be caused by the different milking technique types. Dairy farms with automatic milking systems (AMS) were associated with higher risk of cows showing signs of mastitis compared to dairy farms with conventional milking parlours (*p* < 0.20). AMS are characterized by higher milking frequencies (>2 times/day) and deemed to be a risk factor for udder health [74,76]. The authors stated that udder cleaning processes are conducted in a standardized way without final control of udder cleanliness. Therefore, soiled teats might cause udder infections with environmental pathogens [77]. Furthermore, milking units of the AMS are shared by up to 70 cows. This might lead to higher risks of transmitting pathogens in AMS, compared to conventional milking parlours with several milking units [74,78]. However, it should be mentioned that the distribution of AMS and milking parlours in the present study was unequal (6 AMS v. 36 MP). Therefore, the observed results must be interpreted with caution.

### 4.7. Social Behaviour

Agonistic interactions such as head butts and displacements can be observed mostly in stall areas with high competition for resources such as water trough, feed bunk, cubicles or mechanical brushes [79]. Dairy farms with medium (95–105%) to high (>105%) cow-to-stall ratios were at a higher risk of frequent agonistic interactions (*p* < 0.05). These findings are in accordance with previous studies. For example, Fregonesi et al. [80] determined a curvilinear increase in displacements (0.7, 0.9, 1.6, 2.1, and 1.9 n/5 h) with increasing cow-to-stall ratios (100, 109, 120, 133, and 150%). Similarly, Winckler et al. [14] recorded five times more displacements under overstocked (150%) compared to understocked (75%) housing conditions. Furthermore, higher numbers of agonistic interactions were found in dairy farms with low (<105%) compared to medium (105–118%) and high (>118%) cow-to-feeding place ratios. The results of the present study are contrary to the findings reported in the literature. For example, Huzzey et al. [81] found an increasing number of agonistic behaviours with decreasing feed bunk spaces (0.81, 0.61, 0.41, and 0.21 m/cow). Similarly, Krawczel et al. [82] compared different feed bunk densities (100, 113, 131, and 142%) regarding social behaviour at the feed barrier and determined a continuous increase in displacements between treatments. This unexpected result might be explained by the fact that agonistic interactions in the present study were not only assessed at the feed bunk, but also in other parts of the barn such as walking alleys or resting areas [21]. Competition for limited resources such as cow brushes, water troughs or concentrate feeders, might have influenced the number of agonistic interactions. For example, Val-Laillet et al. [83] determined most displacements at the mechanical brushes when compared to feed bunk and stalls (considering the time spent at the resource). Similarly, de Vries et al. [12] found a positive relationship between the presence of cow brushes and an increasing number of displacements. Competitive behaviour of dairy cows at a concentrate self-feeder were examined by Katainen et al. [84]. The authors reported that nearly half of the visits (42%) at the concentrate feeder were interrupted by butting of other cows. Positive associations between provision of concentrate feeder stations and shorter trough length per cow (<4.7 cm) with higher numbers of agonistic interactions were also found in the present study (*p* < 0.20). In contrast, no association between provision of rotating cow brushes and the results within the criterion “social behaviour” was proven (*p* > 0.20). This might be explained by a sufficient number of cow brushes per group or adequate location within the barn [85].

## 5. Conclusions

The findings of the present study indicate several associations between housing and management factors and selected animal welfare indicators of the WQP for dairy cattle in the univariable statistical analysis. In contrast, only single effects on severe lameness, milk somatic cell count, and social behaviour were determined in multivariable statistical analysis. The lack of associations might be explained by the relatively small sample size of the present study and unbalanced distributions of some potential influencing factors. Under consideration of these limitations, the applied benchmarking approach delivered promising results. Comparing farms with higher and lower animal welfare levels within specific animal welfare indicators regarding selected housing and management factors provided useful information about effective options to improve animal welfare in dairy cattle. However, further research with larger datasets considering different farm types (conventional, organic), housing systems (tie stall, free stall, straw yards) and management options (pasture access, indoor housing) should be conducted to increase the explanatory power of this benchmarking approach.

## Figures and Tables

**Table 1:** Farm characteristics (mean, standard deviation, minimum, maximum) and housing conditions (number of farms, percentage of farms) of 63 dairy cattle farms.

Farm Characteristics ^1^				Housing Conditions			
Parameter	Mean ± SD	Min	Max		Barn Design	Number	Percentage
Herd size [n]	368 ± 346	45	1609	Cubicles	Deep bedded	46	73%
Cows in milk [n]	318 ± 302	41	1353		Rubber mat	17	27%
Group size [n]	99 ± 46	32	237	Floors	Solid	32	51%
Milk yield [kg/cow/a]	9915 ± 943	6870	11,805		Slatted	31	49%
Fat content [%]	4.0 ± 0.2	3.6	4.5	Feeding	Feed rack	30	48%
Protein content [%]	3.4 ± 0.1	3.2	3.6		Neck tube	33	52%
ECM [kg/cow/a]	9880 ± 914	7091	11,747	Insulation	Insulated	32	51%
BTSCC [cells/ml]	249 ± 78	88.0	417.0		Not insulated	31	49%

^1^ Group size = largest number of cows in a group; ECM = energy corrected milk; BTSCC = bulk tank somatic cell count; SD = standard deviation.

**Table 2:** Data collected for the assessment of the animal welfare level of lactating dairy cows using the Welfare Quality^®^ protocol for dairy cattle (modified after Coignard et al. [24]).

Indicator	Frequency Calculation	Method for Collecting Data
Body condition score	Body condition score observed on a sample of cows (N_fin_) ^1^ on the day of the visit	Animal is assessed using the scale: 0—regular body condition **1—very lean body condition** ^2^ 2—very fat body condition
Integument alterations	Prevalence of integument alterations observed on a sample of cows (N_fin_) on the day of the visit	Observation of five body regions (neck/shoulder/back, hindquarter, tarsus, flank/side/udder, carpus) on one side of the animal. On each region, number of hairless patches and lesions/swellings of a minimum diameter of 2 cm are recorded
Lameness (loose house)	Prevalence of lameness observed on a sample of cows (N_fin_) on the day of the visit	Cows are observed when walking on a hard surface. Animal is assessed using the scale: 0—not lame: timing of steps and weight-bearing equal on all four feet 1—moderately lame: imperfect temporal rhythm in stride creating a limp **2—severely lame: reluctance to bear weight on one limb or more than one limb affected**
Milk somatic cell count	Prevalence of cows with subclinical udder inflammation within the last 3 months	Cow milk somatic cell counts are obtained from individual milk records and assessed using the scale: 0—somatic cell count below 400,000 cells/mL for the last 3 months **2—somatic cell count above 400,000 cells/mL at least once in the last 3 months**
Agonistic interactions	Observed in representative segments of the barn on the day of the visit	Recording using continuous behaviour sampling during a total period of 120 min: **Number of head butts, displacements, chasing (-up) and fights**

^1^ N_fin_ = sample size according to [21]; ^2^ Indicators marked in bold were used for statistical analysis.

**Table 3:** Classification of groups with high animal welfare (HW; upper tertile) and low animal welfare (LW; lower tertile) within specific animal welfare indicators or criterion (body condition score, integument alterations, lameness, milk somatic cell count, and social behaviour); levels = thresholds for upper and lower tertiles.

Indicator/Criterion	Group	Level	Mean ^1^	SD	Min	Max
Body condition score (%)	HW	<9.2	6.3	2.3	0.0	9.1
	LW	>15.6	19.9	4.1	15.9	29.2
Integument alterations (%)	HW	<27.1	16.5	6.5	6.3	27.1
	LW	>38.0	47.4	6.9	38.6	62.7
Lameness (%)	HW	<22.0	11.9	6.0	0.0	21.9
	LW	>37.0	52.9	11.7	37.5	74.3
Milk somatic cell count (%)	HW	<18.0	13.0	4.6	2.3	17.6
	LW	>22.5	27.6	4.0	22.8	37.4
Social behaviour (points)	HW	>90.0	92.6	1.9	90.1	96.6
	LW	<83.5	71.6	13.7	40.4	83.0

^1^ Mean, standard deviation, minimum, maximum.

**Table 4:** Potential influencing factors in univariable logistic regression analysis associated with being a herd with high welfare level (HW) within the indicator “body condition score” [Odds ratio, confidence interval, bold *p*-values (*p* < 0.20) were included in multivariable analyses; final model presented at the bottom of the table].

Potential Influencing Factors	Level	LW *	HW *	OR	95% CI	*p*
Feeding frequency (times/day)	1	11	6	0.390	0.108,1.407	**0.150**
	>1	10	14	1	-	
Pushing of feed (times/day)	<4	7	6	0.571	0.108, 3.036	0.717
	4–6	10	8	0.533	0.111, 2.564	
	>6	4	6	1	-	
Calculation of rations (times/year)	<4	7	4	0.571	0.108, 3.036	0.599
	4–11	7	9	1.286	0.286, 5.774	
	>11	6	6	1	-	
Calculation of feed remains	No	5	3	0.565	0.116, 2.758	0.480
	Yes	16	17	1	-	
Body condition scoring (times/lactation)	<1	6	4	0.667	0.129, 3.446	0.803
	1–3	7	8	1.143	0.266, 4.913	
	<4	7	7	1	-	
Feeding regime ^1^	PMR	6	1	0.125	0.868, 73.613	**0.066**
	TMR	15	20	1	-	
Feeding groups (rations)	1	11	4	0.227	0.057, 0.913	**0.037**
	>1	10	16	1	-	
Amount of staple feed (%)	>66	6	3	0.300	0.054, 1.669	0.386
	61–66	4	4	0.600	0.108, 3.338	
	<60	6	10	1	-	
Amount of concentrates (kg/day)	<6.9	6	2	0.111	0.016, 0.778	**0.085**
	6.9–8.5	4	7	0.519	0.104, 2.581	
	>8.5	6	9	1	-	
Cow-to-feeding place ratio (%)	>118	7	4	0.762	0.151, 3.856	0.286
	105–118	6	11	2.444	0.572, 10.447	
	<105	8	6	1	-	
*No significant effects for selected housing and management variables were found in the final model (p > 0.05)*						

* Number of herds included in logistic regression models were partly lower than n = 21, because single housing and management factors were not available for some herds. LW = low welfare group, HW = high welfare group. ^1^ PMR = partial mixed ration, TMR = total mixed ration.

**Table 5:** Potential influencing factors in univariable logistic regression analysis associated with being a herd with high welfare level (HW) within the indicator “integument alterations” [Odds ratio, confidence interval, bold *p*-values (*p* < 0.20) were included in multivariable analyses; final model presented at the bottom of the table].

Potential Influencing Factors	Level	LW *	HW *	OR	95% CI	*p*
Cubicle type ^1^	RM	4	4	1.000	0.214, 4.666	1.000
	DB	17	17	1	-	
Cubicle cleaning (times/day)	1	5	5	1.000	0.123, 8.128	1.000
	2	14	14	1.000	0.098, 10.166	
	3	2	2	1	-	
Mean cubicle width (cm)	<110	8	8	1.800	0.415, 7.814	0.303
	110–113	4	8	3.599	0.710, 18.251	
	>113	9	5	1	-	
Mean cubicle length (cm)	<186	6	3	0.563	0.105, 3.023	0.368
	186–195	6	10	1.875	0.467, 7.526	
	>195	9	8	1	-	
Mean distance neck rail to curb (cm)	<197	7	11	2.750	0.583, 12.976	0.372
	197–205	7	5	1.250	0.233, 6.714	
	>205	7	4	1	-	
Presence of brisket locator	Yes	15	17	1.700	0.402, 7.198	0.471
	No	6	4	1	-	
Mean neck rail height (cm)	<113	6	9	2.000	0.456, 8.777	0.621
	113–119	7	6	1.143	0.250, 5.224	
	>119	8	6	1	-	
Mean feeding place height (cm)	<129	8	3	0.234	0.041, 1.328	0.224
	129–140	8	10	0.781	0.183, 3.342	
	>140	5	8	1	-	
Feeding rack type ^2^	NR	12	9	0.563	0.166, 1.910	0.356
	HL	9	12	1	-	
Feeding place inclined	Yes	9	11	1.467	0.434, 4.951	0.537
	No	12	10	1	-	
*No significant effects for selected housing and management variables were found in the final model (p > 0.05)*						

* Number of herds included in logistic regression models were partly lower than n = 21, because single housing and management factors were not available for some herds. LW = low welfare group, HW = high welfare group. ^1^ RM = rubber mats, DB = deep-bedded, ^2^ NR = neck rail, HL = head lock.

**Table 6:** Potential influencing factors in univariable logistic regression analysis associated with being a herd with high welfare level (HW) within the indicator “lameness” [Odds ratio, confidence interval, bold *p*-values (*p* < 0.20) were included in multivariable analyses; final model presented at the bottom of the table].

Potential Influencing Factors	Level	LW *	HW *	OR	95% CI	*p*
Cow-to-stall ratio (%)	>105	10	4	0.343	0.070, 1.684	**0.131**
	95–105	5	10	1.714	0.371, 7.918	
	<95	6	7	1	-	
Grooves in the floor	No	10	10	1.222	0.353, 4.235	0.752
	Yes	9	11	1	-	
Frequency floor scraping (times/day)	<2	9	6	0.444	0.087, 2.276	0.525
	2–10	5	7	0.933	0.169, 5.151	
	>10	4	6	1	-	
Floor scraping type ^1^	MAN	4	6	1.385	0.312, 6.136	0.668
	AUT	12	13	1	-	
Frequency claw trimming (times/year)	<2.0	13	7	0.179	0.028, 1.136	**0.167**
	2–2.5	6	7	0.389	0.056, 2.697	
	>2.5	2	6	1	-	
Type of claw trimming ^2^	HER	2	4	2.235	0.362, 13.782	
	IND	19	17	1	-	
Person who trims claws ^3^	PRO	1	2	2.105	0.176, 25.166	
	FAR	20	19	1	-	
Footbath routinely used	No	11	1	0.041	0.005, 0.367	**0.004**
	Yes	9	20	1	-	
Flooring type ^4^	SLA	16	9	0.234	0.062, 0.882	**0.032**
	SOL	5	12	1	-	
Rubber on the floors	No	20	13	0.081	0.009, 0.728	**0.025**
	Yes	1	8	1	-	
Access to pasture	No	16	18	1.875	0.385, 9.120	0.436
	Yes	5	3	1	-	
***Final model: r^2^ = 0.10***						
*Footbath routinely used*	*No*	*11*	*1*	*0.043*	*0.005, 0.387*	***0.005***
	*Yes*	*9*	*20*	*1*	*-*	

* Number of herds included in logistic regression models were partly lower than n = 21, because single housing and management factors were not available for some herds. LW = low welfare group, HW = high welfare group. ^1^ MAN = manual, AUT = automatic, ^2^ HER = whole herd, IND = individual cows, ^3^ PRO = professional, FAR = farmer, ^4^ SLA = slatted floor; SOL = solid floor.

**Table 7:** Potential influencing factors in univariable logistic regression analysis associated with being a herd with high welfare level (HW) within the indicator “milk somatic cell count” [Odds ratio, confidence interval, bold *p*-values (*p* < 0.20) were included in multivariable analyses; final model presented at the bottom of the table].

Potential Influencing Factors	Level	LW *	HW *	OR	95% CI	*p*
Type of milking ^1^	AMS	5	1	0.160	0.017, 1.511	**0.110**
	MP	16	20	1	-	
Age of milking equipment (years)	>20	3	6	2.000	0.378, 10.577	0.360
	10–20	9	5	0.556	0.133, 2.325	
	<10	9	9	1	-	
Interim disinfection	No	7	8	1.230	0.347, 4.357	0.748
	Yes	14	13	1	-	
Milking frequency (times/day)	>2	12	5	0.234	0.062, 0.882	**0.032**
	2	9	16	1	-	
Cleaning teats (towels) ^2^	REU	6	5	0.556	0.133, 2.325	0.421
	DIS	10	15	1	-	
Pre-dip routinely	No	17	16	0.376	0.064, 2.224	0.281
	Yes	2	5	1	-	
Post-dip routinely	No	7	5	0.536	0.136, 2.109	0.372
	Yes	12	16	1	-	
Milking sick cows separately	No	12	12	0.750	0.199, 2.827	0.671
	Yes	6	8	1	-	
Fixation after milking	No	11	15	1.591	0.417, 6.073	0.497
	Yes	7	6	1	-	
Dry cow therapy ^3^	DEM	4	10	3.636	0.905, 14.609	**0.069**
	ROU	16	11	1	-	
Intramammary seal	No	2	3	1.500	0.223, 10.076	0.677
	Yes	18	18	1	-	
Udder control during dry period ^3^	No	7	3	0.297	0.060, 1.466	0.329
	DEM	5	5	0.692	0.154, 3.112	
	Yes	9	13	1	-	
***Final model: r^2^ = 0.13***						
*Frequency milking (times/day)*	*>2*	*12*	*5*	*0.208*	*0.054, 0.800*	***0.022***
	*2*	*9*	*16*	*1*	*-*	

* Number of herds included in logistic regression models were partly lower than n = 21, because single housing and management factors were not available for some herds. LW = low welfare group, HW = high welfare group. ^1^ AMS = automatic milking system, MP = milking parlour; ^2^ REU = reusable, DIS = disposable; ^3^ DEM = on demand, ROU = routinely.

**Table 8:** Potential influencing factors in univariable logistic regression analysis associated with being a herd with high welfare level (HW) within the criterion “social behaviour” [Odds ratio, confidence interval, bold *p*-values (*p* < 0.20) were included in multivariable analyses; final model presented at the bottom of the table].

Potential Influencing Factors	Level	LW *	HW *	OR	95% CI	*p*
Regrouping during lactation	Yes	13	15	1.846	0.483, 7.062	0.370
	No	8	5	1	-	
Integration of heifers ^1^ (time)	PP	17	12	0.314	0.078, 1.260	**0.102**
	AP	4	9	1	-	
Cow-to-stall ratio (%)	>105	11	3	0.099	0.018, 0.551	**0.030**
	95–105	6	7	0.424	0.087, 2.061	
	<95	4	11	1	-	
Mean feeding alley width (m)	<3.2	10	3	0.167	0.031, 0.904	**0.082**
	3.2–3.6	6	9	0.833	0.185, 3.750	
	>3.6	5	9	1	-	
Mean walking alley width (m)	<2.4	8	3	0.292	0.056, 1.525	0.231
	2.4–2.7	6	9	1.167	0.279, 4.871	
	>2.7	7	9	1	-	
Mean crossover width (m)	<2.4	7	5	0.446	0.090, 2.215	0.612
	2.4–3.0	7	8	0.714	0.158, 3.231	
	>3.0	5	8	1	-	
Mean walking space ^2^ (m^2^)	<3.7	10	5	0.222	0.045, 1.094	**0.181**
	3.7–4.4	7	7	0.444	0.092, 2.150	
	>4.4	4	9	1	-	
Concentrate feeder station	No	11	17	3.864	0.967, 15.443	**0.056**
	Yes	10	4	1	-	
Rotating cow brush	No	8	9	1.219	0.355, 4.185	0.754
	Yes	13	12	1	-	
Feeding rack type ^3^	NR	11	14	1.818	0.522, 6.331	0.348
	HL	10	7	1	-	
Cow-to-feeding place ratio (%)	>118	7	5	1.786	0.349, 9.127	**0.042**
	105–118	4	12	7.500	1.484, 37.905	
	<105	10	4	1	-	
Trough length per cow (cm)	<4.7	11	4	0.218	0.047, 1.005	**0.092**
	4.7–6.8	4	7	1.050	0.214, 5.158	
	>6.8	6	10	1	-	
***Final model: r^2^ = 0.18***						
*Cow-to-stall ratio (%)*	*>105*	*11*	*3*	*0.099*	*0.018, 0.551*	***0.030***
	*95–105*	*6*	*7*	*0.424*	*0.087, 2.061*	
	*<95*	*4*	*11*	*1*	*-*	

* Number of herds included in logistic regression models were partly lower than n = 21, because single housing and management factors were not available for some herds. LW = low welfare group, HW = high welfare group. ^1^ PP = after calving, AP = before calving; ^2^ excluding lying areas; ^3^ NR = neck rail; HL = head lock.

## Data Availability

Raw data of the study are not deposited in an official storage location. The documents are available on request from the authors.

## References

[1] EFSA (2010). Animal Welfare Risk Assessment Guidelines on Housing and Management. Technical Report (EFSA-Q-2009-00844). Wageningen UR Livestock Research. http://www.efsa.europa.eu/en/supporting/pub/en-87.

[2] Arnott G., Ferris C.P., O’Connell N.E. (2016). Review: Welfare of dairy cows in continuously housed and pasture-based production systems. Animal.

[3] Bewley J.M., Robertson L.M., Eckelkamp E.A. (2017). A 100-Year Review: Lactating dairy cattle housing management. J. Dairy Sci..

[4] Dippel S., Dolezal M., Brenninkmeyer C., Brinkmann J., March S., Knierim U., Winckler C. (2009). Risk factors for lameness in cubicle housed Austrian Simmental dairy cows. Prev. Vet. Med..

[5] Solano L., Barkema H.W., Pajor E.A., Mason S., LeBlanc S.J., Zaffino Heyerhoff J.C., Nash C.G.R., Haley D.B., Vasseur E., Pellerin D. (2015). Prevalence of lameness and associated risk factors in Canadian Holstein-Friesian cows housed in freestall barns. J. Dairy Sci..

[6] Cook N.B., Bennett T.B., Nordlund K.V. (2004). Effect of free stall surface on daily activity patterns in dairy cows with relevance to lameness prevalence. J. Dairy Sci..

[7] Kester E., Holzhauer M., Frankena K. (2014). A descriptive review of the prevalence and risk factors of hock lesions in dairy cows. Vet. J..

[8] Brenninkmeyer C., Dippel S., Brinkmann J., March S., Winckler C., Knierim U. (2013). Hock lesion epidemiology in cubicle housed dairy cows across two breeds, farming systems and countries. Prev. Vet. Med..

[9] Rodrigues A.C.O., Caraviello D.Z., Ruegg P.L. (2005). Management of Wisconsin dairy herds enrolled in milk quality teams. J. Dairy Sci..

[10] Wenz J.R., Jensen S.M., Lombard J.E., Wagner B.A., Dinsmore R.P. (2007). Herd management practices and their association with bulk tank somatic cell count on United States dairy operations. J. Dairy Sci..

[11] Doherr M.G., Roesch M., Schaeren W., Schallibaum M., Blum J.W. (2007). Risk factors associated with subclinical mastitis in dairy cows on Swiss organic and conventional production system farms. Vet. Med..

[12] De Vries M., Bokkers E.A.M., van Reenen C.G., Engel B., van Schaik G., Dijkstra T., de Boer I.J.M. (2015). Housing and management factors associated with indicators of dairy cattle welfare. Prev. Vet. Med..

[13] Collings L.K.M., Weary D.M., Chapinal N., von Keyserlingk M.A.G. (2011). Temporal feed restriction and overstocking increase competition for feed by dairy cattle. J. Dairy Sci..

[14] Winckler C., Tucker C.B., Weary D. (2015). Effects of under- and overstocking freestalls on dairy cattle behavior. Appl. Anim. Beh. Sci..

[15] Espejo L.A., Endres M.I. (2007). Herd-level risk factors for lameness in high-producing Holstein cows housed in freestall barns. J. Dairy Sci..

[16] Lombard J.E., Tucker C.B., von Keyserlingk M.A.G., Kopral C.A., Weary D.M. (2010). Associations between cow hygiene, hock injuries, and free stall usage on US dairy farms. J. Dairy Sci..

[17] Barrientos A.K., Chapinal N., Weary D.M., Galo E., von Keyserlingk M.A.G. (2013). Herd-level risk factors for hock injuries in freestall-housed dairy cows in the northeastern United States and California. J. Dairy Sci..

[18] De Vries M., Bokkers E.A.M., van Schaik G., Engel B., Dijkstra T., de Boer I.J.M. (2016). Improving the time efficiency of identifying dairy herds with poorer welfare in a population. J. Dairy Sci..

[19] Sumner C.L., von Keyserlingk M.A.G., Weary D.M. (2018). How benchmarking motivates farmers to improve dairy calf management. J. Dairy Sci..

[20] Jewell M.T., Cameron M., Spears J., McKenna S.L., Cockram M.S., Sanchez J., Keefe G.P. (2019). Prevalence of hock, knee, and neck skin lesions and associated risk factors in dairy herds in the Maritime Provinces of Canada. J. Dairy Sci..

[21] Welfare Quality® (WQP) (2012). Welfare Quality® Assessment Protocol for Cattle. Welfare Quality® Consortium: Lelystad, Netherlands. http://www.welfarequalitynetwork.net/media/1088/cattle_protocol_without_veal_calves.pdf.

[22] Destatis (2017). Land- und Forstwirtschaft, Fischerei Rinder- und Schweinebestand. Statistisches Bundesamt. Fachserie 3 Reihe 4.1. https://www.destatis.de/DE/Themen/Branchen-Unternehmen/Landwirtschaft-Forstwirtschaft-Fischerei/Tiere-Tierische-Erzeugung/Publikationen/Downloads-Tiere-und-tierische-Erzeugung/viehbestand-tierische-erzeugung-2030400177004.html.

[23] Von Keyserlingk M.A.G., Barrientos A., Ito K., Galo E., Weary D.M. (2012). Benchmarking cow comfort on North American freestall dairies: Lameness, leg injuries, lying time, facility design, and management for high-producing Holstein dairy cows. J. Dairy Sci..

[24] Coignard M., Guatteo R., Veissier I., de Boyer des Roches A., Mounier L., Lehébel A., Bareille N. (2013). Description and factors of variation of the overall health score in French dairy cattle herds using the Welfare Quality^®^ Assessment protocol. Prev. Vet. Med..

[25] De Vries M., Engel B., den Uijl I., van Schaik G., Dijkstra T., de Boer I.J.M., Bokkers E.A.M. (2014). Assessment time of the Welfare Quality^®^ protocol for dairy cattle. Anim. Welf..

[26] Heath C.A.E., Lin Y., Mullan S., Browne W.J., Main D.C.J. (2014). Implementing Welfare Quality^®^ in UK assurance schemes: Evaluating the challenges. Anim. Welf..

[27] Krueger A., Cruickshank J., Trevisi E., Bionaz M. (2020). Systems for evaluation of welfare on dairy farms. J. Dairy Res..

[28] Maroto Molina F., Pérez Marín C.C., Molina Moreno L., Agüera Buendia E.I., Pérez Marín D.C. (2020). Welfare Quality^®^ for dairy cows: Towards a sensor-based assessment. J. Dairy Res..

[29] De Boyer des Roches A., Lardy R., Capdeville J., Mounier L., Veissier I. (2019). Do International Commission of Agricultural and Biosystems Engineering (CIGR) dimension recommendations for loose housing of cows improve animal welfare?. J. Dairy Sci..

[30] Gaworski M., Bockowski M. (2018). Method for comparing current versus recommended housing conditions in dairy cattle production. Agric. Food Sci..

[31] Popescu S., Borda C., Diugan E.A., Niculae M., Stefan R., Sandru C.D. (2014). The effect of the housing system on the welfare quality of dairy cows. Ital. J. Anim. Sci..

[32] Zuliani A., Romanzin A., Corazzin M., Salvador S., Abrahantes J.C., Bovolenta S. (2017). Welfare assessment in traditional mountain dairy farms: Above and beyond resource-based measures. Anim. Welf..

[33] De Boyer des Roches A., Veissier I., Coignard M., Bareille N., Guatteo R., Capdeville J., Gilot-Fromont E., Mounier L. (2014). The major welfare problems of dairy cows in French commercial farms: An epidemiological approach. Anim. Welf..

[34] Benatallah A., Ghozlane F., Marie M. (2015). Dairy cow welfare assessment on Algerian farms. Afr. J. Agric. Res..

[35] De Vries M., Bokkers E.A.M., van Schaik G., Botreau R., Engel B., Dijkstra T., de Boer I.J.M. (2013). Evaluating results of the Welfare Quality multi-criteria evaluation model for classification of dairy cattle welfare at the herd level. J. Dairy Sci..

[36] Armbrecht L., Lambertz C., Albers D., Gauly M. (2019). Assessment of welfare indicators in dairy farms offering pasture at differing levels. Animal.

[37] Haskell M.J., Rennie L.J., Bowell V.A., Bell M.J., Lawrence A.B. (2006). Housing system, milk production, and zero-grazing effects on lameness and leg injury in dairy cows. J. Dairy Sci..

[38] Cook N.B., Hess J.P., Foy M.R., Bennett T.B., Brotzman R.L. (2016). Management characteristics, lameness, and body injuries of dairy cattle housed in high-performance dairy herds in Wisconsin. J. Dairy Sci..

[39] Wagner K., Brinkmann J., Bergschmidt A., Renziehausen C., March S. (2021). The effects of farming systems (organic vs. conventional) on dairy cow welfare, based on the Welfare Quality^®^ protocol. Animal.

[40] Andreasen S.N., Wemelsfelder F., Sandoe P., Forkman B. (2013). The correlation of qualitative behavior assessments with Welfare Quality^®^ protocol outcomes in on-farm welfare assessment of dairy cattle. Appl. Anim. Beh. Sci..

[41] De Graaf S., Ampe B., Tuyttens F.A.M. (2017). Assessing dairy cow welfare at the beginning and end of the indoor period using the Welfare Quality^®^ protocol. Anim. Welf..

[42] DeVries T.J., von Keyserlingk M.A.G., Beauchemin K.A. (2005). Frequency of feed delivery affects the behavior of lactating dairy cows. J. Dairy. Sci..

[43] Sova A.D., LeBlanc S.J., McBride B.W., DeVries T.J. (2013). Associations between herd-level feeding management practices, feed sorting, and milk production in freestall dairy farms. J. Dairy Sci..

[44] Hosseinkhani A., DeVries T.J., Proudfoot K.L., Valizadeh R., Veira D.M., von Keyserlingk M.A.G. (2008). The effects of feed bunk competition on the feed sorting behavior of close-up dry cows. J. Dairy Sci..

[45] Endres M.I., Espejo L.A. (2010). Feeding management and characteristics of rations for high-producing dairy cows in freestall herds. J. Dairy Sci..

[46] Llonch P., Mainau E., Ipharraguerre I.R., Bargo F., Tedo G., Blanch M., Manteca X. (2018). Chicken or the Egg: The Reciprocal Association Between Feeding Behavior and Animal Welfare and Their Impact on Productivity in Dairy Cows. Front. Vet. Sci..

[47] Bewley J.M., Schutz M.M. (2008). Review: An interdisciplinary review of body condition scoring for dairy cattle. Prof. Anim. Sci..

[48] Adams A.E., Lombard J.E., Fossler C.P., Román-Muñiz I.N., Kopral C.A. (2017). Associations between housing and management practices and the prevalence of lameness, hock lesions, and thin cows on US dairy operations. J. Dairy Sci..

[49] Soonberg M., Kass M., Kaart T., Leming R., Arney D.R. (2018). Additional concentrates do not affect feeding times of cows, but social positions of cows do. Agron. Res..

[50] Berry D.P., Veerkamp R.F., Dillon P. (2006). Phenotypic profiles for body weight, body condition score, energy intake, and energy balance across different parities and concentrate feeding levels. Livest. Sci..

[51] Roche J.R., Friggens N.C., Kay J.K., Fisher M.W., Stafford K.J., Berry D.P. (2009). Invited review: Body condition score and its association with dairy cow productivity, health, and welfare. J. Dairy Sci..

[52] Nash C.G.R., Kelton D.F., DeVries T.J., Vasseur E., Coe J., Zaffino Heyerhoff J.C., Bouffard V., Pellerin D., Rushen J., de Passillé A.M. (2016). Prevalence of and risk factors for hock and knee injuries on dairy cows in tiestall housing in Canada. J. Dairy Sci..

[53] Blanco-Penedo I., Ouweltjes W., Ofner-Schröck E., Brügemann K., Emanuelson U. (2020). Symposium review: Animal welfare in free-walk systems in Europe. J. Dairy Sci..

[54] Wechsler B., Schaub J., Friedli K., Hauser R. (2000). Behaviour and leg injuries in dairy cows kept in cubicle systems with straw bedding or soft lying mats. Appl. Anim. Behav. Sci..

[55] Schenkenfelder J., Winckler C. (2022). To meet or not to meet welfare outcome thresholds: A case-control study in dairy cow herds. Animal.

[56] Potterton S.L., Green M.J., Harris J., Millar K.M., Whay H.R., Huxley J.N. (2011). Risk factors associated with hair loss, ulceration, and swelling at the hock in freestall-housed UK dairy herds. J. Dairy Sci..

[57] Kielland C., Ruud L.E., Zanella A.J., Osteras O. (2009). Prevalence and risk factors for skin lesions on legs of dairy cattle housed in freestalls in Norway. J. Dairy Sci..

[58] Ekman L., Nyman A.-K., Landin H., Persson Waller K. (2018). Hock lesions in dairy cows in freestall herds: A cross-sectional study of prevalence and risk factors. Acta Vet. Scand..

[59] Zaffino Heyerhoff J.C., LeBlanc S.J., DeVries T.J., Nash C.G.R., Gibbons J., Orsel K., Barkema H.W., Solano L., Rushen J., de Passillé A.M. (2014). Prevalence of and factors associated with hock, knee, and neck injuries on dairy cows in freestall housing in Canada. J. Dairy Sci..

[60] Tucker C.B., Jensen M.B., de Passillé A.M., Hänninen L., Rushen J. (2021). Invited review: Lying time and the welfare of dairy cows. J. Dairy Sci..

[61] Falk A.C., Weary D.M., Winckler C., von Keyserlingk M.A.G. (2012). Preference for pasture versus freestall housing by dairy cattle when stall availability indoors is reduced. J. Dairy Sci..

[62] Webster A.J.F. (2001). Effects of housing and two forage diets on the development of claw horn lesions in dairy cows at first calving and in first lactation. Vet. J..

[63] Oehm A.W., Knubben-Schweizer G., Rieger A., Stoll A., Hartnack S. (2019). A systematic review and meta-analyses of risk factors associated with lameness in dairy cows. Vet. Res..

[64] Chapinal N., Barrientos A.K., von Keyserlingk M.A.G., Galo E., Weary D.M. (2013). Herd-level risk factors for lameness in freestall farms in the northeastern United States and California. J. Dairy Sci..

[65] Rouha-Mülleder C., Iben C., Wagner E., Laaha G., Troxler J., Waiblinger S. (2009). Relative importance of factors influencing the prevalence of lameness in Austrian cubicle loose-housed dairy cows. Prev. Vet. Med..

[66] Griffiths B.E., Grove White D., Oikonomou G. (2018). A Cross-Sectional Study into the Prevalence of Dairy Cattle Lameness and Associated Herd-Level Risk Factors in England and Wales. Front. Vet. Sci..

[67] Ariza J.M., Levallois P., Bareille N., Arnoult A., Guatteo R. (2020). Short communication: Evaluation of a foot dirtiness scoring system for dairy cows. J. Dairy Sci..

[68] Solano L., Barkema H.W., Pickel C., Orsel K. (2017). Effectiveness of a standardized footbath protocol for prevention of digital dermatitis. J. Dairy Sci..

[69] Nyman A.-K., Ekman T., Emanuelson U., Gustafsson A.H., Holtenius K., Persson Waller K., Hallén Sandgren C. (2007). Risk factors associated with the incidence of veterinary-treated clinical mastitis in Swedish dairy herds with a high milk yield and a low prevalence of subclinical mastitis. Prev. Vet. Med..

[70] Barkema H.W., Schukken Y.H., Lam T.J.G.M., Beiboer M.L., Benedictus G., Brand A. (1999). Management practices associated with the incidence rate on clinical mastitis. J. Dairy Sci..

[71] Kabera F., Roy J.-P., Afifi M., Godden S., Stryhn H., Sanchez J., Dufour S. (2021). Comparing Blanket vs. Selective Dry Cow Treatment Approaches for Elimination and Prevention of Intramammary Infections During the Dry Period: A Systematic Review and Meta-Analysis. Front. Vet. Sci..

[72] Ruegg P.L. (2017). A 100-Year Review: Mastitis detection, management, and prevention. J. Dairy Sci..

[73] Zecconi A., Gusmara C., Di Giusto T., Cipolla M., Marconi P., Zanini L. (2020). Observational study on application of a selective dry-cow therapy protocol based on individual somatic cell count thresholds. Ital. J. Anim. Sci..

[74] Hovinen M., Pyörälä S. (2011). Invited review: Udder health of dairy cows in automatic milking. J. Dairy Sci..

[75] Smith J.W., Ely L.O., Graves W.M., Gilson W.D. (2002). Effect of milking frequency on DHI performance measures. J. Dairy Sci..

[76] Jacobs J.A., Siegford J.M. (2012). Invited review: The impact of automatic milking systems on dairy cow management, behavior, health, and welfare. J. Dairy Sci..

[77] Dohmen W., Neijenhuis F., Hogeveen H. (2010). Relationship between udder health and hygiene on farms with an automatic milking system. J. Dairy Sci..

[78] Barkema H.W., von Keyserlingk M.A.G., Kastelic J.P., Lam T.J.G.M., Luby C., Roy J.-P., LeBlanc S.J., Keefe G.P., Kelton D.F. (2015). Invited review: Changes in the dairy industry affecting dairy cattle health and welfare. J. Dairy Sci..

[79] Foris B., Thompson A.J., von Keyserlingk M.A.G., Melzer N., Weary D.M. (2019). Automatic detection of feeding- and drinking-related agonistic behaviour and dominance in dairy cows. J. Dairy Sci..

[80] Fregonesi J.A., Tucker C.B., Weary D.M. (2007). Overstocking reduces lying time in dairy cows. J. Dairy Sci..

[81] Huzzey J.M., DeVries T.J., Valois P., von Keyserlingk M.A.G. (2006). Stocking density and feed barrier design affect the feeding and social behavior of dairy cattle. J. Dairy Sci..

[82] Krawczel P.D., Klaiber L.B., Butzler R.E., Klaiber L.M., Dann H.M., Mooney C.S., Grant R.J. (2012). Short-term increases in stocking density affect the lying and social behavior, but not the productivity, of lactating Holstein dairy cows. J. Dairy Sci..

[83] Val-Laillet D., Veira D.M., von Keyserlingk M.A.G. (2008). Short communication: Dominance in free-stall-housed dairy cattle is dependent upon resource. J. Dairy Sci..

[84] Katainen A., Norring M., Manninen E., Laine J., Orava T., Kuoppala K., Saloniemi H. (2005). Competitive behavior of dairy cows at a concentrate self-feeder. Acta Agric. Scand. A Anim. Sci..

[85] Goncu S., Yesil M.I., Yilmaz N. (2019). The Cattle Grooming Behavior and Some Problems with Technological Grooming Instruments for Cow Welfare. J. Environ. Sci. Engin. B.

